# Development of an internet-delivered cognitive behavioral therapy program for use in combination with exercise therapy and education by patients at increased risk of chronic pain following total knee arthroplasty

**DOI:** 10.1186/s12913-021-07177-7

**Published:** 2021-10-25

**Authors:** Turid Rognsvåg, Maren Falch Lindberg, Anners Lerdal, Jan Stubberud, Ove Furnes, Inger Holm, Kari Indrekvam, Bjørn Lau, Daniil Rudsengen, Søren T. Skou, Mona Badawy

**Affiliations:** 1 Coastal Hospital in Hagevik, Department of Orthopedic Surgery, Haukeland University Hospital, Hagaviksbakken 25, N-5217 Hagavik, Norway; 2 Department of Clinical Medicine, University of Bergen, Bergen, Norway; 3 Faculty of Medicine, Institute of Health and Society, Department of Nursing Science, Oslo, Norway; 4 Department of Research, Lovisenberg Diaconal Hospital, Oslo, Norway; 5 Faculty of Medicine, Department of Interdisciplinary Health Sciences, University of Oslo, Oslo, Norway; 6 Department of Psychology, University of Oslo, Oslo, Norway; 7 The Norwegian Arthroplasty Register, Department of Orthopedic Surgery, Haukeland University Hospital, Bergen, Norway; 8 Orthopedic Surgery, Oslo University Hospital, Oslo, Norway; 9 Research Unit for Musculoskeletal Function and Physiotherapy, Department of Sports Science and Clinical Biomechanics, University of Southern Denmark, Odense, Denmark; 10 The Research Unit PROgrez, Department of Physiotherapy and Occupational Therapy, Næstved-Slagelse-Ringsted Hospitals, Slagelse, Denmark

**Keywords:** Osteoarthritis, Total knee arthroplasty, Cognitive behavior therapy, Physical exercise

## Abstract

**Background:**

Approximately 20% of patients experience chronic pain after total knee arthroplasty (TKA). Due to the growing number of TKA procedures, this will affect an increasing number of people worldwide. Catastrophic thinking, dysfunctional illness perception, poor mental health, anxiety and depression characterize these non-improvers, and indicate that these patients may need individualized treatment using a treatment approach based on the bio-psycho-social health model. The present study developed an internet-delivered cognitive behavioral therapy (iCBT) program to be combined with exercise therapy and education for patients with knee osteoarthritis (OA) at increased risk of chronic pain after TKA.

**Methods:**

The development process followed the first two phases of the UK Medical Research Council framework for complex interventions. In the development phase, the first prototype of the iCBT program was developed based on literature review, established iCBT programs and multidisciplinary workshops. The feasibility phase consisted of testing the program, interviewing users, condensing the program, and tailoring it to the patient group. A physiotherapist manual was developed and adapted to physiotherapists who will serve as mentors.

**Results:**

The development process resulted in an iCBT program consisting of 10 modules with educational texts, videos and exercises related to relevant topics such as goalsetting, stress and pain, lifestyle, automatic thoughts, mindfulness, selective attention, worry and rumination. A physiotherapist manual was developed to guide the physiotherapists in supporting the patients through the program and to optimize adherence to the program.

**Conclusions:**

The iCBT program is tailored to patients at risk of chronic pain following TKA, and may be useful as a supplement to surgery and/or exercise therapy. A multicentre RCT will evaluate the iCBT program in combination with an exercise therapy and education program. This novel intervention may be a valuable contribution to the treatment of OA patients at risk of chronic pain after TKA.

**Trial registration:**

The RCT is pre-registered at ClinicalTrials.gov: NCT03771430 11/12/2018.

## Background

Total knee arthroplasty (TKA) for osteoarthritis (OA) is quite a successful procedure, with improvements in pain, function and quality of life [1, 2]. However, studies consistently show that 20% of patients have questionable benefit from TKA and continue to experience pain and poor function without clinical explanation [3, 4] and without any effective treatments available [5]. The incidence of TKA procedures worldwide is growing [6], with more than 700,000 procedures annually in the United States alone [7], and is estimated to increase by 143% by 2050 [8]. Thus, TKA non-responders represent a large and growing number of patients who continue to suffer from unrelieved pain and poor function [9, 10]. Consequently, they are less likely to return to work and more likely to be high consumers of health care services [11–13]. Current treatment modalities for knee OA are based on the Osteoarthritis Research Society International (OARSI) recommendations for evidence-based treatment, which include education, exercise, lifestyle alterations, weight loss when relevant, and analgesics [14]. The effectiveness of exercise is comparable to that of Non-Steroidal Anti-Inflammatory Drugs (NSAIDs), with effects lasting at least 2 to 6 months [15]. Patients with moderate to severe OA who do not benefit from non-surgical interventions may be considered candidates for TKA surgery. A recent study by Skou and colleagues tested a non-surgical treatment program based on the OARSI recommendations alone or as postoperative follow-up after TKA. While the TKA group had larger improvements in pain and function over time, the non-surgical group also showed clinically relevant improvements. Only 26 and 32% of them decided to undergo surgery 12 and 24 months after the intervention, respectively [1, 16]. These results demonstrate the beneficial impact of non-surgical interventions on OA symptoms.

However, the OARSI-based treatment modalities alone may not be sufficient for all patients. A growing literature suggests that non-improvers following TKA have a distinct preoperative psychological profile characterized by catastrophic thinking [17], dysfunctional illness perception [4], poor mental health [18], anxiety [19] and depression [20]. These factors may hamper engagement in physical activity and rehabilitation due to pain-related fear of movement or motivational problems [21, 22]. Such factors can represent a pathway that may cause a poor outcome following TKA surgery. As such, these patients may need individualized treatment using a more comprehensive treatment approach based on the bio-psycho-social health model [23].

In cognitive behavioral therapy (CBT), pain is recognized as a complex, subjective phenomenon, and the use of CBT in the management of chronic pain thus fits well with the bio-psycho-social health model [24, 25]. Research has shown that, whether administered alone or in combination with medical or interdisciplinary rehabilitation treatment, CBT improved pain and related problems in chronic pain patients [26, 27]. The gate control theory [28], although not correct in detail [29], forms the basis of psychological treatment of pain and emphasizes the importance of cognitive and affective, as well as sensory, influences on pain. The premise for CBT in relation to pain is to identify and modify pain-enhancing thinking patterns, or cognitions, maladaptive behavior and situations that contribute to the maintenance of psychological distress, which may lead to further progression of pain [30]. The aim of CBT utilization is to reduce pain and psychological distress, in addition to increasing adaptive behaviors such as participation in exercises and day-to-day activities. A CBT protocol developed by Turk et al. [31] addresses a number of psychological factors that may impact pain intensity and disability, such as catastrophic thinking [32, 33], fear-avoidance [34], low self-efficacy, helplessness and lack of perceived control [35–38], in addition to passive pain coping strategies [39]. Among these, pain-related catastrophic thinking and pain-related fear had the strongest associations with pain intensity and disability in patients with knee OA [40]. Various pain coping skill programs have shown promise in OA patients [41–44] and can be effectively delivered as internet-based CBT [45].

Our research team aimed to take these results one step further and develop an evidence-based and internet-delivered CBT (iCBT) program for all OA patients who are candidates for TKA, but specifically targeted for patients less likely to benefit from standard TKA treatment. The program was designed to be combined with an exercise therapy and education program based on AktivA [46], consisting of a 90-min patient education session followed by exercise therapy twice a week for 12 weeks. To support patients and enhance the treatment’s effects, specially trained physiotherapists will also serve as patient mentors throughout the program. Based on this prior evidence, we expect that such a combined program may result in better treatment outcomes for the large and growing number of non-responders after TKA surgery.

The aims of the present research were to:

1. Develop an iCBT program to be combined with an exercise therapy and education program for patients with knee OA at increased risk of chronic pain after TKA (Phase 1)
2. Thoroughly test and customize the program (Phase 2)

## Methods

This paper originates from the MultiKnee multi-center randomized controlled trial (pre-registered at ClinicalTrials.gov: NCT03771430 11/12/2018), investigating the effectiveness of an exercise therapy and education program combined with iCBT on pain and functional outcomes in patients with higher risk of chronic pain following TKA. The development process is presented according to guidance for reporting intervention development (GUIDED) [47].

**Fig. 1:**
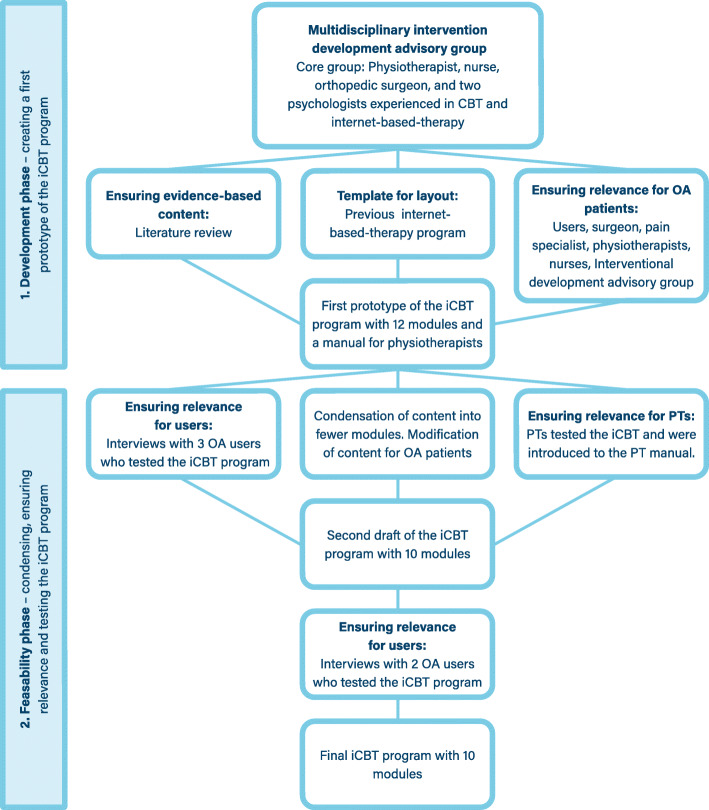
Flow chart describing the process of developing the electronic version of a Cognitive Behavioral Theory-based (iCBT) intervention

The UK Medical Research Council (MRC) framework for complex interventions [48] served as a foundation for the program’s development process. The MRC framework is a stepwise approach that focuses largely on preliminary groundwork to optimize the development of complex interventions. The framework is flexible and consists of distinct, but iterative phases. First, the development phase was used to identify the evidence base and theory, and model underlying pathways. Secondly, a feasibility phase was performed with input from users and clinicians. The stages in the development of the iCBT intervention is presented in Fig. 1. The program is based on general principles for CBT [24] and adapted to reflect causes and treatment of OA pain and pain after TKA surgery. A literature search was performed to ensure that the program was grounded in current evidence. Furthermore, OA patients’ opinions of the program were sought through individual user interviews.

### Phase 1: development phase – creating a first prototype of the iCBT program

A multidisciplinary intervention development advisory group was established. The group was broadly composed of national and international representatives consisting of nurses (*n* = 3), physiotherapists (*n* = 3), orthopedic surgeons (*n* = 4), psychologists (*n* = 2), a pain specialist and a health economist, all with long-term experience in clinical practice and research. The group met regularly to identify and define the topic and discuss theoretical and practical questions. Furthermore, a core group consisting of a physiotherapist, a nurse and an orthopedic surgeon with long-term experience from the TKA field, in addition to two psychologists with extensive experience in CBT and internet-based therapy, were responsible for designing the iCBT program.

#### Literature review

To identify the available evidence, a literature review was conducted on the following topics (results in parenthesis):

- Guidelines for the management of OA patients [14, 49].
- Psychological interventions in OA and TKA patients [41, 42, 44, 50–52].
- Internet-based CBT interventions for OA and TKA patients [44, 45, 53–56].
- The relationship between psychological factors and pain in OA and TKA patients [35, 36, 57–59].

Discussions in the advisory group and results from the literature review formed the rationale, theory and goal for the intervention and the selection of included elements.

#### Rationale, theory and goal

The core treatment for knee OA is exercise therapy combined with education and weight reduction if needed [14]. OA patients may face significant challenges in initiating and maintaining these treatments in the long term. Barriers to physical activity and exercise may include pain during exercise, low self-efficacy, depressive symptoms, anxiety, feeling of helplessness, and low social support or activity [21, 22]. Some of these factors are also shown to be predictors of poor outcome after TKA [60]. However, using CBT, these barriers may be reduced by developing more adaptive cognitions and behaviors. Consequently, adherence to exercise and physical activity may be improved [25]. Importantly, because physical activity and psychological treatment methods likely have synergistic effects, adding iCBT to exercise therapy and education may result in better treatment outcomes [26, 27]. The core premise of CBT is that maladaptive cognitions contribute to the maintenance of emotional distress and behavioral problems. Hence, in CBT a variety of techniques are combined in order to develop more adaptive cognitions and behaviors, including psychoeducation, cognitive restructuring, relaxation therapy and guided imagery (e.g. reduce muscle tension and autonomic arousal), mindfulness training, problem-solving, and stress management [24, 25]. In particular, in the context of pain, CBT focuses on reducing pain and distress by modifying physical sensations, catastrophic and ruminative thinking, and maladaptive behaviors [30], in addition to enhancing self-efficacy [35, 36].

Hence, the goal of the intervention is to increase patients’ awareness of their own thoughts and behavior, and to learn and practice new ones so they can initiate, maintain or resume their normal physical and social activities. Further learning goals are to increase patients’ confidence in making their own assessments and to learn techniques for dealing with pain in an appropriate way.

#### Template for layout

The first draft of the iCBT program was designed from relevant elements of the commercially-available Braive program [61], which is based on well-documented treatment principles.

#### Relevance for OA patients

Since the iCBT program elements from Braive were not specifically designed for OA patients, it was necessary to tailor and adjust the content by emphasizing OA pain and cognitions associated with OA pain. Two versions of the iCBT program were developed, one non-surgical version for OA patients, and one version for patients undergoing TKA surgery. A persona, an animated figure based on a typical OA or TKA patient, was created for each version of the program. The personas represent a figure that OA or TKA patients can identify with, and appear in all modules throughout the programs. To help patients see the relevance of the iCBT exercises in each module and how to implement them into their exercise therapy program, the iCBT exercises in both versions included lists of relevant examples for OA and TKA patients. Both versions were identical in content except for minor differences in the examples and personas. The interventional development advisory group, users and physiotherapists were consulted and contributed their input throughout development of the program. This phase yielded a prototype of an iCBT program with 12 modules and a manual for physiotherapists serving as patient mentors.

### Phase 2: feasibility phase – condensing, ensuring relevance and testing the iCBT program

In this stage, we evaluated whether the program was relevant, manageable and understandable for the patients and whether the program and the clinician manual were relevant for the physiotherapists. This process was characterized by feedback-loops where users and researchers were challenged to give input to refine the program.

#### Relevance for patients

To evaluate the program’s relevance for OA patients, and its feasibility and acceptability, we conducted interviews with users in two rounds. For planning and conducting the interviews, Norman and Skinner’s eHealth literacy model [62] was employed. Of particular interest were users’ experiences with navigating the program, understanding the information and instructions, and appraising the usefulness of the program for the target group.

The first draft of the program was distributed to three users, two men and one woman, who had undergone TKA surgery, followed by individual interviews conducted by a physiotherapist. The interview guide and results from the first round of interviews are described in Table 1. Two of them were positive to the program and would have joined if given the opportunity. Their input was used to improve the program, and resulted in a more condensed and manageable iCBT program. Consequently, the examples and information pages became more relevant and understandable to the patients. When the second draft of the iCBT program was completed, a second round of interviews was conducted with two of the same users. The results (Table 2) were used to further refine the revised version of the program. The conclusion from the user interviews was that the iCBT program would be useful for many patients as a supplement to surgery and/or exercise therapy.

**Table 1:** Inteview round 1

Interview guide:	Results:
What are your immediate thoughts on this program now that you have seen an overview of all the modules?	*“This seems exciting. I liked the video about Kathrine, recognized myself in her story.”* *“I oppose this “dehumanization”. I am in favor of personal contact and that not everything should happen online. I think many, especially the elderly, will have trouble completing the course because lack of computer knowledge.”* *“Exciting, I would have been keen on it!”*
Is the content per module manageable to complete in 1 week?	“*… manageable …*” *“… too extensive, takes a lot of time”* *“… .may seem overwhelming to some, important to only get one module a week”*
Is the presentation understandable? Words, expressions etc.	*“… change some expressions...”* *“… very good information, some information becomes too philosophical … want more specific information related to osteoarthritis”* *“..some of the terms are incomprehensible, some bad wording and bad language …”*
Are the examples recognizable?	*“the story about Kathrine is recognizable … some of the other examples should be changed to make them more recognizable to osteoarthritis patients”* *“… some examples become incomprehensible for osteoarthritis patients”* *“… some of the examples do not fit this patient group”*
Will this cause the patients to get the spikes out thinking that we think “it is only in your head” or that we do not take their pain seriously?	*“… important to emphasize that physiotherapy is the main element of this intervention”* *“… clarify how thoughts, attitudes and stress affect pain”* *“… I don’t think the module about values is relevant, and can be provocative, must either be removed or come later in the program …*. I *also perceive the module on Rest Networks more as psychotherapy … can be provocative for this patient group”*
What do you think about the level / difficulty of the content - easy to follow or advanced?	*“… easy to follow, manageable”* *“The program is too comprehensive … too difficult for many due to lack of computer skills”* *“I had trouble logging into the program the first time … it was easy to navigate in the program … intuitive and easy to know where to press to move forward … the layout and ease of use is good … the hand that drew the drawings was disturbing … still image would have been better.”*
Would you be willing to do this if you were told that you were in the target group?	*… would think this was exciting* *… would not join … no need for “everything” to take place on the internet”* *“… this seemed exciting, I would want to join”*

**Table 2:** Interview round 2

	Interview guide:	Results:
Find	How was it to log into and navigate in the program?	*“no problem”*
	Is the content per module manageable to complete in 1 week?	*“no problem”* *“some of the modules are demanding, important that the patients are prepared, suggest to divide into two parts”*
Understand	How is the presentation? Words, expressions etc.	*“good explanations, understandable”* *“they talk too fast, suggestion: work through the sequences twice and more”* *“some typos”*
	How were the exercises? Did you understand what to do?	“*OK exercises”* *“some of the exercises are demanding, suggest to split them”*
	Are the examples recognizable?	*“have not seen the examples”* *“good examples, there is a possibility that patients will copy the examples instead of thinking what is relevant for them”*
	What do you think about the level / difficulty of the content - easy to follow or advanced?	“*the level of difficulty is OK”* *“some of the modules and exercises are demanding”*
Apprise	How relevant is the content for you as an OA/TKA^a^ patient?	*“good program as part of a larger context”* *“good program, important to emphasize that the rehabilitation period lasts for several months”*
	How will the content impact the users? Will this cause the patients to think that we do not take their pain seriously?	*“unsure if it is too optimistic and moralizing, important to emphasize that it is part of a larger package”*
Useful	How useful will this program be for you?	*“useful as a supplement following the operation”* *Useful to manage day to day life”*
	How useful do you think this program will be for others?	*“I think this program will be useful for many patients”*

^a^ *OA* Osteoarthritis, *TKA* Total knee arthroplasty

#### Condensation of content

To discuss further condensation of the content, the professionals in the research group arranged a workshop. The aim was to tailor the program to the patient group and condense it to the most essential CBT elements. Priorities were made based on the literature [31], feedback from user interviews and knowledge about the patient group. The condensation included a reduction of modules from 12 to 10. Topics such as goal setting, relaxation techniques, mindfulness and worry and rumination were prioritized, while content related to values, core beliefs, and rules and assumptions for living, in addition to body scan and autogenic training, were omitted.

#### Ensuring relevance for physiotherapists

A physiotherapist manual was developed in order to ensure treatment fidelity. Four physiotherapists experienced in treating patients undergoing TKA surgery were introduced to the iCBT program and the physiotherapist manual to optimize their relevance and usefulness. A workshop was arranged where the physiotherapists discussed the relevance and feasibility of all elements of the manual. Revisions were made accordingly, such as clarification of the physiotherapist’s role and customization of the information sheet.

Phase 2 resulted in a final version of the iCBT program consisting of ten modules (Table 3), accompanied by a physiotherapist manual (Table 4) containing a brief introduction to CBT and basic motivational interviewing (MI) [63] techniques, in addition to instructions for each module. The iCBT program and manual are presented in detail in the Results section.

**Table 3:** Overview of the content in each of the 10 sessions of the cognitive-behavioral intervention

Session	Theme	Content	Exercise	Theory and goal
1.	Getting started	• Gate control theory (video) • Learn to know Kathrine (video) • The relation between thoughts, feelings and behavior (video) • Relaxation technique	• Try the relaxation technique • Writing exercise: Life Story	• Knowledge about pain mechanisms and the interaction of thoughts, emotions and behavior form the basis of change • Learn relaxation technique to reduce muscle tension and autonomic arousal
2.	Goals for the recovery	• Five key elements important for coping with pain (medical, mental wellbeing, lifestyle, life story, physical activity) (video) • FAQ physical activity • Follow Kathrine • Goals for recovery	• Make a pie chart; important areas to focus on • My goal for recovery • Writing exercise: Affirmative Writing • Reminder: relaxation technique	• Awareness of how it is possible to cope with pain form the basis of changing unhelpful behavior • Knowledge about physical activity reduce fear-avoidance behavior • Goalsetting increase motivation and adherence to the program
3.	Stress and pain	• How to change habits (video) • Understanding and managing stress (video) • Identifying main stressors • Locus of control (video)	• Identifying main stressors • Writing exercise: How has pain affected you? • Update goals for recovery • Reminder: relaxation technique	• Understanding stress, how to change habits and locus of control promotes changing processes • Reflective practice to increase awareness of own stressors
4.	Lifestyle	• How different kind of lifestyle can contribute to the symptoms (training and restitution) (video) • How worry and anxiety influence behavior (video) • Safety behavior (video)	• Identify and challenge safety behavior • Writing exercise: Safety behavior and lifestyle • Update goals for recovery • Reminder: relaxation technique	• Knowledge about how lifestyle factors, worry and anxiety influence behavior, can motivate to change behavior • Be aware of own safety behavior and challenge it to start the process of changing behavior
5.	Identifying automatic thoughts	• Thinking errors (video) • How challenging situations can be perceived as threat, loss or challenge (video) • The inner dialogue (video)	• Exploration of internal dialogue • Writing exercise: Pain triggers and alternative thoughts • Update goals for recovery • Reminder: relaxation technique	• Education about thinking errors and internal dialogue to start reflecting on own thoughts • Use the writing exercise to raise awareness about pain triggers and generate alternative thoughts
6.	Creating alternative thoughts	• Common thinking errors (video)	• Identify thinking errors and generate alternative thoughts • Writing exercise: Emotional expression • Update goals for recovery • Reminder: relaxation technique	• Practice in identifying thinking errors and generating alternative thoughts to continue the process of changing thoughts and behavior
7.	Be more mindful	• Default Mode Network (DNM) and mental habits (video) • Focused attention (video) • Conscious refocusing (audio file)	• Practice conscious refocusing • Writing exercise: Going Deeper • Update goals for recovery • Reminder: relaxation technique	• Practice focused attention and conscious refocusing to reduce DNM activity
8.	Selective attention	• Becoming more mindful (video) • Selective attention (video)	• Mindfulness exercise: “Floating leaves” (audio file) • Writing exercise: Choose perspective • Update goals for recovery • Reminder: relaxation technique	• Practice guided imagery and selective attention to reduce muscle tension and autonomic arousal
9.	Postponing worry and rumination	• Worry and rumination • Why worry escalates • Postponing worry and rumination • Postponement log	• Make worry postponement log • Writing exercise: Living with loss and changes • Update goals for recovery • Reminder: relaxation technique	• Learn about worry and rumination. • Practice making a worry postponement log • Reflecting on how loss and changes in life affect you, and how to live with it
10.	What’s next?	• What have I learned? • What’s next?	• Writing exercise: What have I learned	• Reflection on what that has been learned and future plans

**Table 4:** Physiotherapist Manual

Week	Theme	Topics to address	Learning goals
1	Get started	• Help patient to get started • Ask if they have tried the relaxation technique • Ask if they have completed diary exercise.	• Learn about the relation between thoughts, feelings and behaviour • Learn a relaxation technique
2	Goals for the recovery	• Ask if the patient has started to fill in pie chart and the Goal podium. • Remind about relaxation technique and writing exercise.	• Be able to support patient in setting goals and using strategies to cope with pain
3	Stress and pain	• Discuss what they consider to be their main stressors • Help to fill in the goal podium and reminder about relaxation techniques and writing exercise.	• Learn about stress and pain, and be able to support patients to change habits
4	Lifestyle	• Ask if the patient has completed the exercise about “safety behaviour” • Help to revise the goal podium • Remind about relaxation techniques and writing exercise.	• Learn about safety behaviour and be able to help patient to be aware of how different kinds of lifestyles can contribute to symptoms
5	Identifying automatic thoughts	• Discuss how it was to do the exercise about “Inner dialogue” • Remind about writing exercise: Pain triggers and alternative thoughts. • Remind about relaxation techniques	• Be able to help patient to be aware of their own thinking errors and automatic thoughts
6	Creating new thoughts	• Ask about what he/she gets out of the information about thinking errors • Ask what experiences he/she had when identifying their own thinking errors • Remind about writing exercise: Emotional expression • Remind about relaxation techniques	• Be able to support patient to identify their own thinking errors and create alternative thoughts
7	Becoming more mindful	• Ask if patient experiences having selective attention directed against threat and loss in relation to their OA • Ask what experiences he/she has in relation to the exercise “conscious refocusing” • Remind about the writing exercise: Going deeper • Remind about relaxation techniques	• Learn about Default Mode Network (DNM) and mental habits to be able to support the patient to become more mindful
8	Selective attention	• Ask patient what they think about the exercise “Floating leaves” • Remind about the writing exercise: Choose Perspective • Remind about relaxation techniques	• Learn about selective attention and be able to support the patient to be more mindful
9	Postponing worry and rumination	• Ask patient if he/she can distinguish between worry and rumination • Ask if he/she can postpone the worry and rumination by creating a “Postponement log” • Remind about writing exercise: Living with loss and changes in life • Remind about relaxation techniques	• Learn about worry, rumination and why worry escalates. Be able to support patient to postpone worry and rumination and make a postponement log
10	What next?	• Discuss what the patient has learned, what he/she has achieved and what remains. • Encourage the patient to look back on previous exercises. • Remind about writing exercise: What have I learned • Discuss what to do next	• Be able to support the patient to use what they have learned and to create new goals in life.
11	Specialization for physiotherapists	• Understanding the concept • The learning model • Key elements in CBT • Home exercises	• Increase physiotherapist’s knowledge about the elements of the intervention
12	Conversation with the participants	• Motivating interview (MI) (video) • MI techniques (video) • Resistance • When users experience challenges • Getting stuck in unhelpful thoughts – encourage meta-perspective • Pitfalls in building alliances • Unhelpful assumptions	• Improve the quality of the interaction between the physiotherapist and the participant

## Results

### Description of the iCBT program

The iCBT program is presented according to the template for intervention description and replication (TIDieR) checklist and guide [64]. To use the iCBT program, participants must have access to the internet and an electronic device (computer, tablet or smartphone). The program will be delivered as a guided, tailored iCBT program in ten modules to be distributed over 10 weeks as shown in Table 3. Patients will be given access to the program through a secure website using two-factor authentication, where they will be introduced to the program and receive further instructions.

#### The iCBT user interface

The iCBT program consists of ten modules. Participants are encouraged to complete one module before moving on to the next. Each module follows a similar structure, consisting of psychoeducational texts and videos that present relevant topics for the module. Most of the modules include a video where the patients can follow the “persona” – the fictional character with OA or TKA, who undergoes either non-surgical treatment (version A) or TKA surgery (version B). Each module includes tasks related to the topics covered. Some tasks can be performed immediately (e.g. writing exercise, relaxation exercise); others are expected to be done as behavioral experiments between the modules.

The purpose of the first two modules is to help patients change their habits and lifestyles, and set new goals in areas that are important for pain management. Based on various psychoeducational texts and videos, patients are challenged to identify areas in which they want to change, and to set step-by-step goals for how the goals can be reached. Throughout the program, patients are challenged to continue to revise their goals in the subsequent sessions by describing the strategies they chose to apply and the progress they have made, and by setting additional goals for their rehabilitation.

#### Physiotherapist manual

To optimize adherence to the program, physiotherapists will support the patients through telephone contact every second week. Using physiotherapists as mentors is intended to facilitate integration of the iCBT and exercise therapy, and increase the likelihood of generalization to daily life. The physiotherapists will participate in a one-day course, led by an experienced psychologist, to be able to support the patients throughout the program. The course includes an introduction to the iCBT program and the physiotherapist manual, in addition to education about CBT principles. The physiotherapist manual will support the physiotherapists and increase the consistency of mentoring the patients. The physiotherapist manual contains the same ten modules from the iCBT program, specific learning objectives for each module, and a list of themes the physiotherapist should consider discussing with the patients, including recommendations as to how each theme might be addressed. In addition, two extra learning modules are available for the physiotherapists. The first module contains an introduction to basic CBT and MI principles. The second module provides guidance on how to handle patients’ resistance and address challenges (Table 4). Furthermore, if the patient grants permission, the physiotherapists can access a secure website to monitor each patient’s progress, and provide support and assistance when necessary.

#### Theoretical content and psychoeducation

The cognitive-behavioral model focusing on the “cognitive diamond”, which illustrates the link between thoughts, emotions, bodily reactions and behavior [24], is the theoretical framework for the program. The model is represented through texts, videos, iCBT exercises, and behavioral experiments throughout the program. For example, the CBT model hypothesizes that when exposed to a stressful situation or condition, such as pain, our self-image and perception of the world tend to become negatively biased. Thus, at the beginning of the program, participants learn to identify negative automatic thoughts and beliefs that arise in painful situations. They are then introduced to how those thoughts can be challenged and modified. In later modules, participants learn about various forms of thinking errors, safety behaviors, internal dialogue, perceiving challenging situations as threats, losses or challenges, locus of control, stressful situations [24, 25, 65], and the gate control theory of pain [28]. At a later session, participants are introduced to a metacognitive theoretical view of worry and rumination [25]. It is explained how worry tends to escalate, and participants learn how to create a postponement log for both worrying and rumination.

#### iCBT exercises

Some iCBT exercises are carried out throughout the program. A diary writing exercise is introduced at the beginning of the program, and patients are asked to write on different topics in the coming sessions. They are also introduced to a relaxation technique (progressive muscle relaxation) and are encouraged to practice it regularly. In a later module, they learn about mindfulness, including selective attention and conscious refocusing, and undergo an exercise in mindfulness that they are encouraged to use repeatedly [66].

## Discussion

In the present paper, we have described the development process of an iCBT program for knee OA and TKA patients at increased risk of chronic pain after TKA surgery, to provide clinicians and researchers with enough details to replicate the program. The developmental process following the MRC framework resulted in an iCBT program consisting of ten modules and a manual to guide the physiotherapists mentoring the patients.

One in five patients undergoing TKA have limited or no effect of the surgery when it comes to pain and function [5]. They are characterized by having one or more psychological factors that may contribute to increased pain and reduced quality of life [40]. CBT aims to help participants develop more adaptive cognitions and behavior [31]. Combined with an evidence-based exercise therapy and education program, we hypothesize iCBT will lead to less pain, better function and improved quality of life for these patients. The evaluation of the effectiveness of the combined program will be performed in a randomized controlled trial.

We base our study on current evidence suggesting that several of the risk factors for a suboptimal TKA outcome are modifiable (e.g., catastrophic thoughts about pain, pain-related anxiety, generalized anxiety, and depression). Because these psychological factors, combined with pain, may constitute significant barriers to participation in exercise therapy [21], we expect that by modifying the risk factors, patients may increase their adherence to exercise and physical activity. Furthermore, exercise can also have a positive effect on mental health [67]. Therefore, as found in patients with hip and knee OA [42, 43], a biopsychosocial approach that combines psychological and physical interventions might produce the best outcome [31].

CBT-based treatment for persons at risk of poor outcome following TKA has been evaluated in several recent studies, which concluded that CBT alone is likely insufficient to improve TKA outcomes [68–70]. While the CBT programs in these prior studies consist of basic CBT elements relatively similar to our study, they were not combined with an individually tailored exercise therapy and education program. Our program builds on these prior studies by combining iCBT with CBT-trained physiotherapists who serve as mentors to help patients integrate their new skills both in the exercise therapy sessions and in daily life. Our iCBT program is also specifically adapted for OA and TKA patients and it has two versions, one for OA patients in general and one specific to patients undergoing TKA.

Because the program is intended to be combined with an exercise therapy and education program, physiotherapists will mentor the patients through the program. Thus, the physiotherapists’ manual was designed to clarify and support the role of the physiotherapists. Using physiotherapists as mentors is in line with findings from a recent study [44], which demonstrated that patients achieved better functional outcome when physiotherapists combined exercise with Pain Coping Skills training compared to either treatment alone. Accordingly, we expect that the combined psychological intervention and exercise therapy mentored by physiotherapists will optimize patients’ results. Using trained physiotherapists as mentors is designed to help patients integrate their skills from the iCBT to cope with pain during their exercise therapy.

This study is the first to create an iCBT program for patients with knee OA and patients undergoing TKA, to be combined with exercise therapy and education. In a recent systematic review, Calbring et al. demonstrated that iCBT targeting psychiatric and somatic conditions is as effective as face-to-face treatment for all conditions studied [53]. For our patient group, iCBT has only been tested in a smaller randomized controlled trial of 69 participants. O’Moore [55] found that a 10-week iCBT depression program effectively reduced depression, and improved self-efficacy, pain, stiffness, and physical function in patients with OA and severe depression.

Internet-based CBT programs have been evaluated in other populations with chronic pain [71, 72] and have shown promising results. However, these prior programs are largely self-directed, requiring minimal, if any, clinician involvement. In contrast, the target population for our iCBT program consists of patients at risk of poor TKA outcome, and these patients may benefit from more clinician involvement to stay motivated and to integrate their new skills both in the exercise program and in daily life. Although our iCBT program consists of many of the same CBT elements as prior studies, our program is uniquely tailored to OA and TKA patients and is specifically designed to be combined with exercise therapy and mentored by specially trained physiotherapists.

Schuster et al. [73] listed several advantages of iCBT. Bridging geographic distances was one of them. Participants in the present study may potentially save time and money participating in iCBT compared to face-to-face therapy. For those patients who have recently undergone surgery, it is an advantage not to travel long distances to a therapist. Another advantage is that they can work through the program and materials whenever it suits them.

However, using online treatment programs may be challenging for patients without internet access or for those who are unfamiliar with using a computer or smartphone. It is therefore likely that the program is more applicable to younger patients who are familiar with using tablets, smartphones or computers. However, the user interface of our program is designed to be as simple and intuitive to use as possible and the program is supported by mentor physiotherapists, which may limit potential barriers to using such a program.

It is estimated that 85% of research activity is wasted [74]. The strength of our work is that it has followed the first two phases in the MRC framework for developing complex interventions. Bleijenberg et al. [75] stated that improving the development of complex interventions “would reduce research waste and enhance the likelihood of success”, and recommended adding four elements to the MRC framework: 1) problem identification and definition, 2) determination of recipients’ and providers’ needs, 3) examination of current practice and context, and 4) intervention design. These elements have been taken into account in our study through the detailed work of the multidisciplinary intervention development advisory group and the core group, consisting of clinicians, researchers and users with extensive experience from the field, representing both recipients and providers.

## Conclusions

We have developed an iCBT intervention tailored to patients at risk of chronic pain following TKA. The development process followed the first two phases of the MRC framework for complex interventions. The iCBT program consists of 10 modules with educational texts, videos and exercises related to relevant topics. A physiotherapist manual guides physiotherapists in mentoring patients through the program. A planned multi-centre three-armed RCT will test the effectiveness of iCBT combined with an exercise therapy and education program.

The iCBT intervention developed in this study may be a valuable contribution to the treatment of knee OA. It is easy to use and less time-consuming for patients and therapists than face-to-face programs. The result of the RCT may contribute to the general knowledge of how to treat patients at risk of an unfavorable TKA outcome. The intervention may benefit a substantial number of patients every year, as well as society by reducing costs associated with chronic pain.

## Data Availability

All data generated or analysed during this study are included in this published article.

## Abbreviations

CBT: Cognitive Behavioral Therapy
GUIDED: Guidance to reporting intervention development
iCBT: Internet-delivered Cognitive Behavioral Therapy
MI: Motivational Interviewing
MRC: Medical Research Council
NSAID: Non-Steroidal Anti Inflammatory Drugs
OA: Osteoarthritis
OARSI: Osteoarthritis Research Society International
RCT: Randomized Controlled Trial
TIDieR: Template for Intervention Description and Replication
TKA: Total Knee Arthroplasty
